# Editorial: Novel therapeutic target and drug discovery for neurological diseases

**DOI:** 10.3389/fphar.2022.1042266

**Published:** 2022-10-07

**Authors:** Kaiyue Zhao, Zhuorong Li, Qingshan Liu, Yong Cheng, George E. Barreto, Rui Liu

**Affiliations:** ^1^ Institute of Medicinal Biotechnology, Chinese Academy of Medical Sciences and Peking Union Medical College, Beijing, China; ^2^ College of Pharmacy, Minzu University of China, Beijing, China; ^3^ College of Life and Environmental Sciences, Minzu University of China, Beijing, China; ^4^ Department of Biological Sciences, University of Limerick, Limerick, Ireland

**Keywords:** Alzheimer’s disease, dementia, depression, drug target, natural products, neurological disorders, neurotrophic factor, stroke

The Research Topic “*Novel Therapeutic Target and Drug Discovery for Neurological Diseases*” consists of 20 articles, including 12 original research papers, seven reviews, and one systematic review, with contributions from more than 140 authors. This work aims to compile a collection of articles focused on recent advances in promising therapeutic targets and biomarkers, new pathological mechanisms, and novel therapeutic agents in order to provide valuable clues for developing new therapeutic strategies for neurological diseases.

One group of included articles discusses targets with potential applications for drug discovery and therapeutic strategies. One such target is the Sigma-1 receptor, a mitochondrion-associated endoplasmic reticulum membrane protein active in the central nervous system, whose antagonists showed attractive potential in treating neuropathic pain and psychostimulant abuse (Hayashi and Su, 2007; Yano et al., 2018). Ren et al. provide a systematic overview of the role of the Sigma-1 receptor in regulating neurotransmission, including regulation of excitatory and inhibitory (E/I) balance *via* glutamatergic, serotonergic, and GABAergic neurotransmitters, suggesting that Sigma-1 receptor agonists may be a potent therapeutic target for depression, particularly in the development of fast-acting antidepressants. Neuraminidase 1 (NEU1) is a subtype of the sialidase family responsible for removing sialic acid residues from protein-bound oligosaccharides. Notably, mutation of NEU1 results in sialidosis, a rare genetic metabolic disease (Miyagi and Yamaguchi, 2012). Khan et al. discuss the associations of NEU1 with neurological disorders and underscore its role in amyloid precursor protein (APP) and amyloid beta-peptide (Aβ) accumulation and Toll-like receptor (TLR) activation-dominated microglial activation in Alzheimer’s disease (AD). The researchers conclude that NEU1 is an emerging therapeutic target for AD. Genomic analysis and transcriptome profiling have recently been used as a hypothesis-free approach to identify novel drug targets for neurological diseases (Zeng et al., 2021; Jiang et al., 2022; Sun et al., 2022). Using RNA sequencing analyses and experimental verification, Zhao et al. identify differentially expressed genes, microRNAs (miRNAs), and transcription factors (TFs), constructed miRNA-TF-gene regulatory networks, and propose several new molecules, such as miR-145-5p and cysteine and serine-rich nuclear protein 1 (*Csrnp1*), as potential therapeutic targets for vascular dementia.

Another group of articles advances our understanding of the pathological mechanisms of neurological diseases. AD is the primary factor leading to dementia with unclear pathogenesis. Islam et al. reveal the specific roles of environmental metals in aberrant protein misfolding and neuroinflammation in the brains of multiple AD animal models, implicating cerebral metabolic disorder as an essential feature of AD. Dai et al. compare the metabolic properties of AD animal models and patients based on 78 metabolomic profiles from the public available data. They propose two proteins, namely Erb-B2 receptor tyrosine kinase 2 (HER2) and neurogenic differentiation factor 2 (NDF2), as promising biomarkers of AD along with 16 metabolic pathways common to AD in mice and patients, implying close associations with the etiology of AD. Cerebrovascular events are also important causes of neurological disorders and often lead to secondary tissue injury; however, there are currently few effective therapeutic treatments (Rost et al., 2022). Mo et al. highlight the roles of the Wnt/catenin signaling pathway in diverse cell types of neurovascular units, including maintaining blood-brain barrier integrity, reducing neuroinflammation and synapse damage, and promoting remyelination, and suggest this pathway as a potential therapeutic target for ischemic stroke. Due to the critical role of microglia in maintaining immune-inflammatory homeostasis, neuroinflammation is closely associated with neurological disorders (Schwartz and Deczkowska, 2016; Colonna and Butovsky, 2017). Wu et al. address the pathological process driven by microglial pyrosis in neurological diseases and present advances in immunological strategies for treating neuroinflammation by targeting the nod-like receptor family pyrin domain containing 3 (NLRP3), caspase-1, and gasdermins (GSDMs).

Several articles cover drug discovery for nervous system diseases. Research related to small molecule entities with specific targets has long attracted attention for AD. Yang et al. report that the N-[N-(3, 5-difluorophenylacetyl)-l-propanoyl]-s-phenylglycine butyl ester (DAPT), an inhibitor of γ-secretase, attenuates cadmium-induced multi-organic damage and cognitive impairment in mice. Furthermore, they report that the neuroprotective effect of DAPT against cadmium toxicity might be associated with inhibition of the Notch/HES-1 signaling axis. Natural products are significant sources of leading compounds in drug research and development. Li et al. report that LY-01, which is derived from the Chinese herbal medicine *Sophora alosecuroides*, alleviates early cognitive decline in 5×familial AD (5×FAD) mice (10 or 13 weeks old) by promoting endogenous neural regeneration. They demonstrate that LY-01 exerts neuroprotective functions by increasing the number of new cells, neuronal precursor cells, and length of neurites in the dentate gyrus of 5×FAD mice, and has similar effects on promoting proliferation of primary neurons, astrocytes, and primary NSCs *in vitro*. Lang et al. report the therapeutic effects of *Coeloglossum viride* var. *bracteatum* extract (CE) on the 1-methyl-4-phenyl-1,2,3,6-tetrahydropyridine (MPTP)-induced mouse model of Parkinson’s disease (PD). CE, which was extracted from tubers of *Coeloglossum viride* var. *bracteatum* (a traditional Tibetan medicine), shows beneficial effects on behavioral disability in mice with MPTP injury. Further investigation of the substantia nigra and striatum of CE-treated mice revealed inhibition of astrocyte activation and neuroinflammation as well as increased neuronal survival *via* recovery of the BDNF-TrkB and FGF2-Akt signaling pathways. Edaravone dexborneol is a novel drug approved in China for the treatment of acute ischemic stroke. Chen et al. report the neuroprotective effect and underlying mechanism of edaravone dexborneol in a rat model of subarachnoid hemorrhage (SAH). They demonstrate that edaravone dexborneol contributes to sensorimotor functions in rats after SAH, inhibits neuronal apoptosis in the affected hippocampus and basal cortex, alleviates oxidative stress, and specifically reduces toxic lipid peroxide-4-hydroxynonenal (4-HNE) levels in neurons and astrocytes. Agathisflavone, a flavonoid with anti-neuroinflammatory and myelinogenic properties, is demonstrated by do Nascimento et al. to protect injured spinal cord tissue by increasing the expression of neurotrophins and modulating the inflammatory response. Mangiferin, which exerts a wide range of pharmacological activities, is shown by Yan et al. to potentially alleviate postpartum depression-like behaviors in mice by inhibiting microglial activation and neuroinflammation *via* mitogen-activated protein kinase (MAPK) signaling pathways. Other studies demonstrate how a structure-based drug discovery strategy contributes to innovative drug development for neurological diseases. For example, by analyzing the low-temperature electromagnetic structure of human tryptophan hydroxylase 2 (TPH2) in the tetrameric state of 3.0 Å using cryoelectron microscopy and testing small molecule activators by molecular docking and molecular dynamics simulation, Zhu et al. suggest that CMPD1 is a potentially promising TPH2-targeted compound for mental disorders.

In summary, the current Research Topic includes original studies, reviews, and a systematic review regarding novel therapeutic targets, molecular mechanisms, and potential drug candidates for the treatment of neurological diseases. The use of advanced technologies such as transcriptomics, network pharmacology, and structural biology technology can help shed light on the complex etiology of central nervous system diseases, identify new targets, and develop novel active substances. Furthermore, natural products with ideal neuropharmacological activity are expected to be developed from substances in fundamental research to translational medicine. Lastly, we extend our sincere gratitude to the Editorial Office of Frontiers in Pharmacology, all of the authors, and all of the reviewers for their valuable contributions to this Research Topic.
